# Development of a statistically standardized optical digital wrist model through integrated MRI-diffuse optical imaging methodology

**DOI:** 10.1117/1.JBO.30.12.126003

**Published:** 2025-12-03

**Authors:** Tong Zhang, Lingxiu Xing, Wenjing Sun, Mai Dan, Wenxing Bai, Jiao Li, Dongyuan Liu, Limin Zhang, Feng Gao

**Affiliations:** a Tianjin University, College of Precision Instrument and Optoelectronics Engineering, Tianjin, China; b Tianjin Key Laboratory of Biomedical Detecting Techniques and Instruments, Tianjin, China

**Keywords:** magnetic resonance imaging, time-domain diffuse optical tomography, spatial frequency domain imaging, digital wrist template, optical digital wrist model

## Abstract

**Significance:**

Current optical health-sensing devices rely on simplified homogeneous tissue models or semi-empirical ratiometric methods, which inadequately address anatomical complexity and inter-individual optical variability. This introduces systematic errors in light propagation modeling, compromising measurement accuracy and clinical robustness, necessitating organ-specific optical models for reliable physiological sensing.

**Aim:**

To develop a standard optical digital wrist (DW) model by integrating magnetic resonance imaging (MRI) and diffuse optical imaging (DOI), enabling anatomically accurate and optically realistic modeling of wrist tissues for improved precision in wearable optical health monitoring applications.

**Approach:**

The multimodal MRI-DOI framework was implemented, comprising three key components: (1) statistical integration of high-resolution MRI datasets generated a population-averaged anatomical DW template; (2) region-based time-domain diffuse optical tomography (TD-DOT) with MRI-derived anatomical priors, extracted depth-resolved optical properties of subsurface tissues; (3) spatial frequency domain imaging (SFDI) supplemented high-resolution optical properties of superficial skin layers.

**Results:**

Simulation experiments demonstrated the high accuracy of region-based TD-DOT reconstruction, with mean errors below 8.57% ($\mu_{a}$) and 9.63% ($\mu_{s}^{\prime }$), quantitatively supporting the precision of the proposed approach. Phantom experiments with wrist-mimicking phantoms yielded mean reconstruction errors of 10.52% ($\mu_{a}$) and 13.23% ($\mu_{s}^{\prime }$) for TD-DOT, and the SFDI top-layer quantification yielded lower errors of 4.48% ($\mu_{a}$) and 8.69% ($\mu_{s}^{\prime }$), validating the performance of the TD-DOT system and the SFDI system. Furthermore, *in vivo* optical property measurements showed strong agreement with literature values, further validating the reliability and practicality of the methodology.

**Conclusions:**

We establish a standard DW template and develop an *in vivo* optical structure acquisition methodology, transitioning biosensing models from homogeneous approximations to anatomically layered models. The approach can enhance the customization, dynamic adaptability, and clinical validity of biosensing technologies.

## 1 Introduction

Optical health-sensing technologies are widely used for non-invasive monitoring of critical physiological parameters, including blood oxygen saturation, heart rate, and hemoglobin concentrations. However, current methods rely on oversimplified geometric models that assume optical homogeneity in biological tissues,^1^^,^^2^ overlooking anatomical heterogeneity and individual optical variability. This leads to systematic measurement biases that limit clinical applicability. Addressing this challenge requires standardized digital organ templates that reflect population-level anatomy, together with practical frameworks for *in vivo* optical characterization.

Magnetic resonance imaging (MRI) provides an excellent basis for template development, offering high-resolution imaging without ionizing radiation and with excellent soft-tissue contrast.^3^^,^^4^ For optical property measurement, time-domain diffuse optical tomography (TD-DOT), a modality of diffuse optical imaging (DOI), enables three-dimensional (3D) simultaneous reconstruction of the absorption and scattering coefficients.^5^^–^^7^ However, clinical adoption faces hurdles due to the ill-posed nature of conventional pixel-based DOT reconstruction, compounded by limited source-detector configurations and complex organ anatomy.^8^^–^^13^ Although prior studies have used MRI-derived prior information to enhance TD-DOT reconstruction reliability and fidelity,^14^ an issue of application is that it is neither economical nor practical for individualized MRI-based structural data acquisition. Furthermore, such strategies remain insufficient for accurately characterizing the optical properties of superficial skin tissues.

Here, we propose an integrated MRI–DOI framework to construct a statistically standardized optical digital organ model. Specifically, we establish a standard digital wrist (DW) template from MRI-derived anatomy then register the standard DW to individual geometry for region-based TD-DOT reconstruction. Compared with conventional voxel-based TD-DOT, this approach partitions the reconstruction domain into tissue-specific subregions, reducing the number of unknowns, alleviating the inverse problem ill-posedness, and improving both spatial resolution and quantitative accuracy.^13^^,^^15^ Complementarily, spatial frequency domain imaging (SFDI) is applied to measure superficial skin properties, offering wide-field, non-contact acquisition and high sensitivity to thin-tissue structures.^16^^–^^19^ Together, this dual-modality strategy bridges deep and superficial tissue characterization, advancing the field from simplified homogeneous or semi-empirical models to anatomically accurate, multilayered models. In addition, this work selected three commonly used and clinically relevant NIRS wavelengths,^20^ 670, 830, and 905 nm, to systematically analyze the optical properties of wrist tissues. The 670 and 830 nm ranges are typically used for assessing superficial tissue hemodynamics,^21^ whereas 905 nm, with lower absorption and greater penetration, is suitable for probing deeper structures such as muscle and bone.^22^ The combination enables non-invasive, depth-resolved measurement of wrist optical properties, providing a valuable reference for biophotonic sensing technologies and enhancing their personalization, adaptability, and clinical applicability.

## 2 Methods

**Fig. 1:**
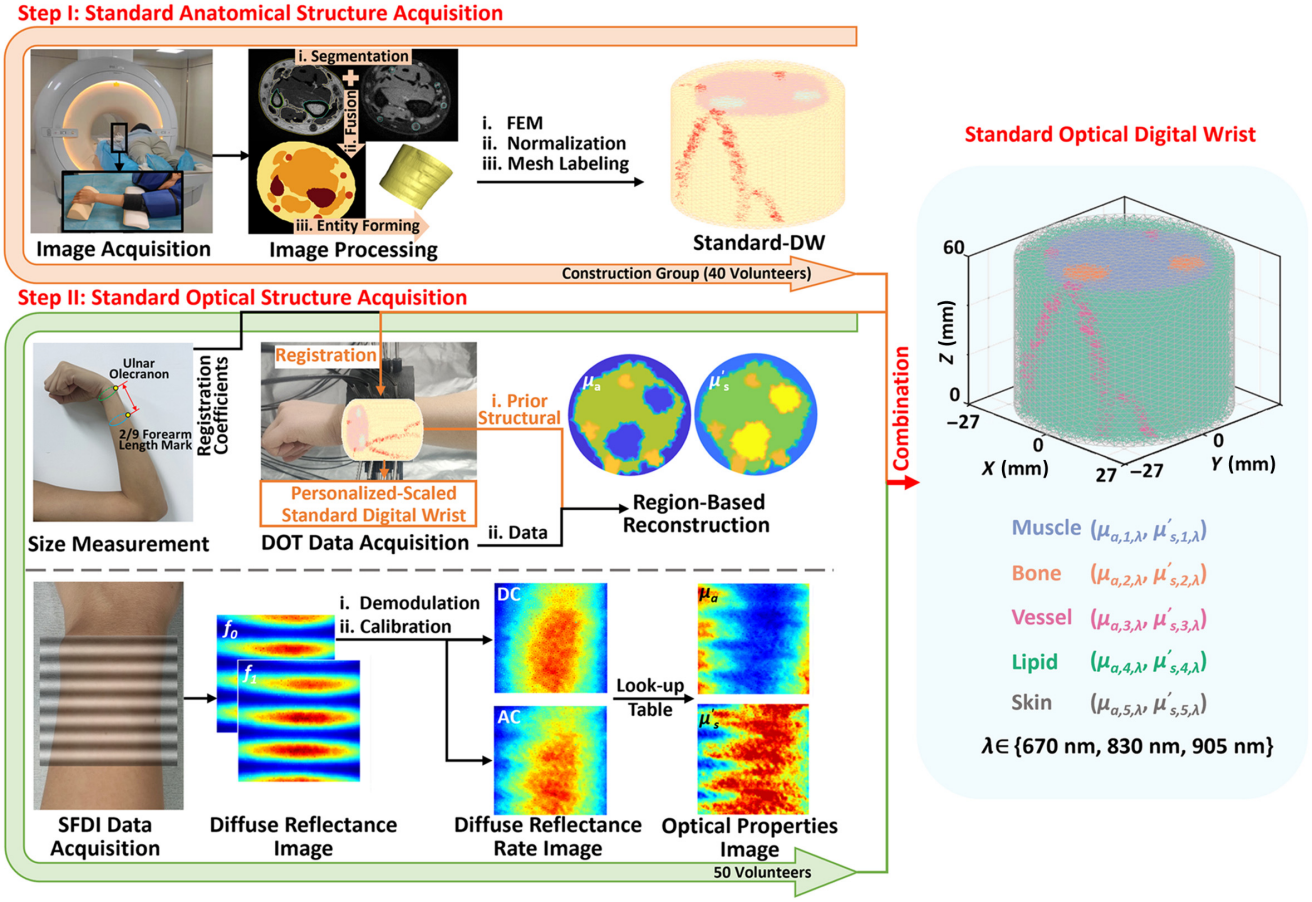
Flowchart of standard optical DW construction.

Figure 1 illustrates the two critical steps required for standard optical DW construction: MRI-based acquisition of standardized anatomical structures and region-based TD-DOT/SFDI for optical characterization of tissues. The work included 50 volunteers (age range: 20 to 60 years) without wrist pathologies and with diverse body types, approved by the Ethics Committee of Tianjin University, with written informed consent obtained before experimentation. Volunteers were divided into two subgroups: a construction group (20 males/20 females) for standard DW template development and a testing group (5 males/5 females) for efficacy assessment, collectively enabling standard optical DW development.

### 2.1 Standard Anatomical Structure Acquisition Using MRI

The standard DW construction includes image acquisition and processing to construct original DWs, followed by comprehensive dimensional normalization to ensure consistency. Statistical analysis of node-related tissue types was performed to complete the standard DW template, and the discrepancies between the standard DW and actual anatomical structures were subsequently evaluated.

#### 2.1.1 Image acquisition and processing

##### Image acquisition

**Fig. 2:**
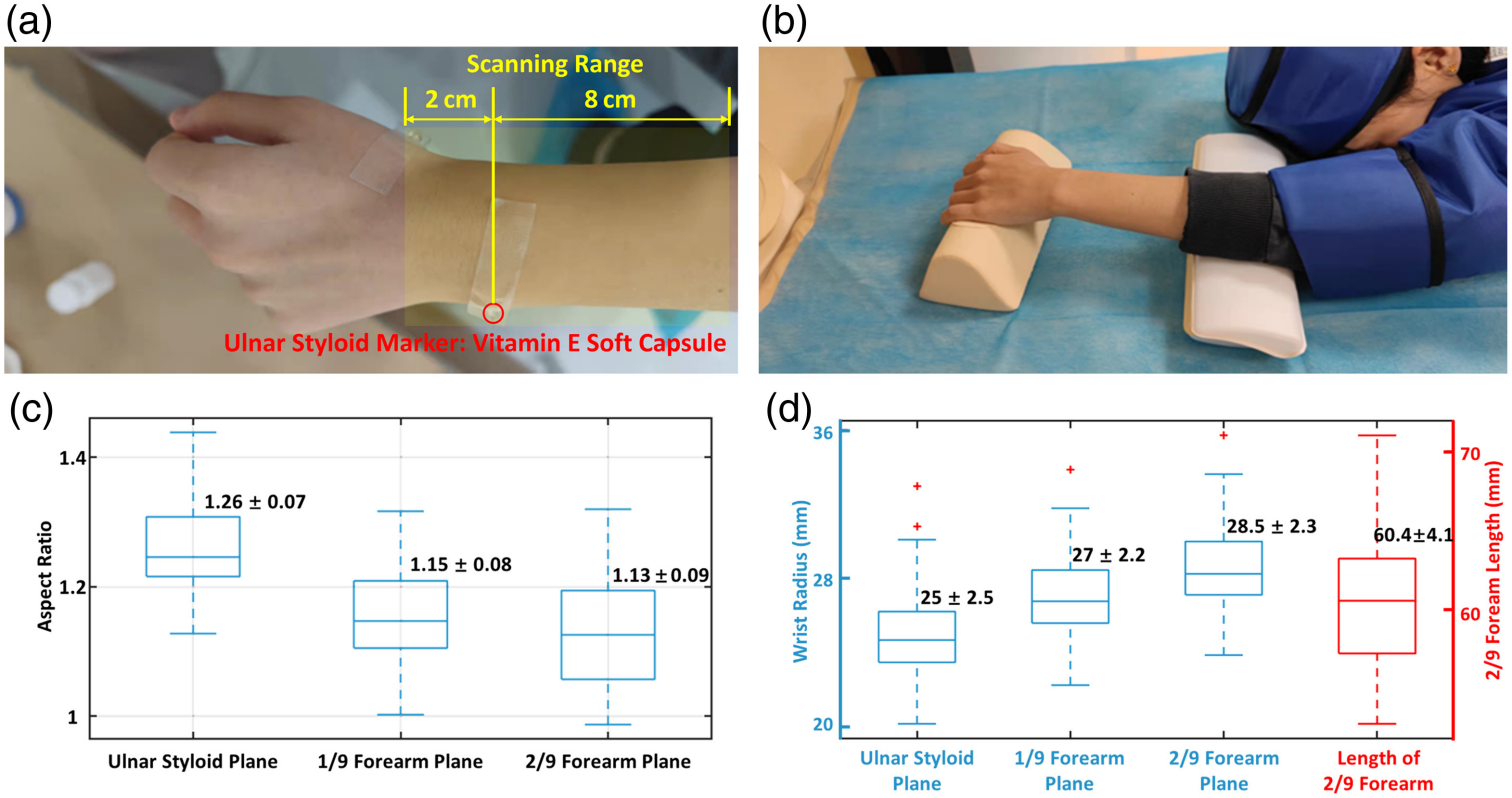
MRI experimental configuration and wrist information. (a) Marker point. (b) Posture. (c) Wrist aspect ratio (major-to-minor axis ratios) statistics. (d) Wrist size statistics.

The ulnar styloid process, an anatomical landmark that is easily identifiable across most individuals, was designated as the reference origin for MRI segmentation. A 4-mm vitamin E soft capsule was placed at the ulnar styloid to serve as a marker [Fig. 2(a)]. It exhibited high signal intensity on both T1-weighted and proton-density-weighted sequences. To accommodate the health-sensing range, the MRI scanning range was set to 8-cm (proximal) and 2-cm (distal) separation from the ulnar styloid, as shown in Fig. 2(a). To ensure the wrist was fixed during MRI scanning, as shown in Fig. 2(b), the volunteer adopted the prone position, with the left arm parallel to the body, the left palm facing downward, and the wrist maintained straight. T1-weighted imaging was performed with a repetition time (TR) of 698 ms, the interval between successive RF pulses on the same slice, and an echo time (TE) of 20 ms, the interval from the RF excitation pulse to the peak of the echo signal. This sequence provides a clear visualization of soft tissue structures, whereas proton density-weighted imaging (TR = 3000 ms, TE = 20 ms) was acquired for bright vascular display. The matrix size was 252×248  pixels, with a field of view of 100×100×110  mm. The slice thickness was set to 2.5 mm, with a slice spacing of 0.725 mm. The images were saved in DICOM format and imported into Amira (Visage Imaging, Richmond, Australia) for image processing.

##### Image processing

The wrist consists of seven major tissues^23^: skin, lipids, muscle, tendons, vasculature, nerves, and bone. Considering MRI’s limited ability to resolve epidermis and nerves and the optical similarity between tendons and muscle,^24^^,^^25^ the segmentation protocol excluded neural structures, amalgamated tendons with muscle, and simply set the superficial 1.5 mm as skin type as described in Sec. 2.1.2. For vascular segmentation, only the radial/ulnar arteries and the cephalic/basilic veins were retained. To ensure consistency among models, the MRI slice range was defined as 2/9 of each volunteer’s forearm length. Previous anatomical studies have confirmed that this range fully encompasses the wrist region,^26^ which not only satisfies the device positioning requirements but also reduces redundant slices. In addition, the ulnar styloid process—the most easily identifiable bony landmark of the wrist—was selected as the starting reference for image segmentation.

**Fig. 3:**
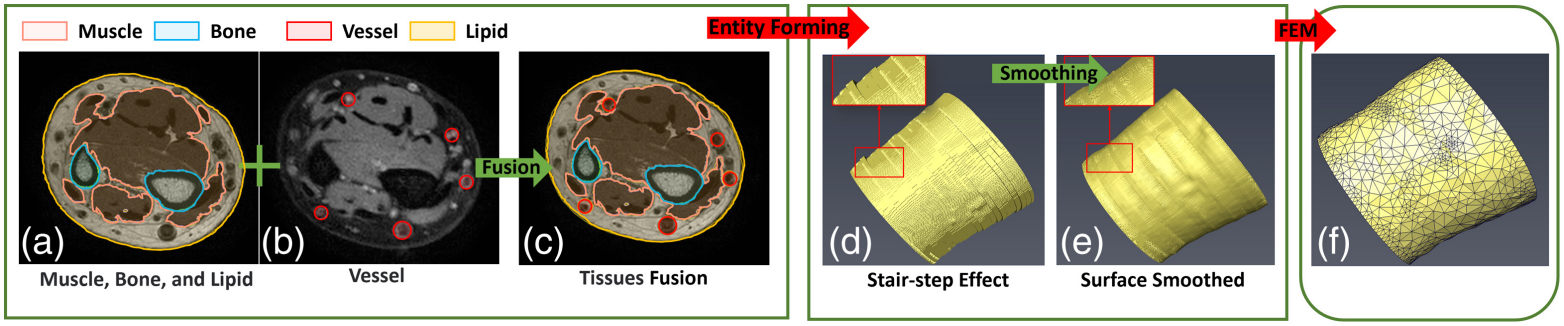
(a)–(f) Flowchart of original DW generation.

Image segmentation, fusion, and others were performed using the relevant functions of the Amira, employing a semi-automatic processing approach.^27^ Initial tissue differentiation (muscle, adipose, and bone) from T1-weighted sequences employed grayscale threshold-based segmentation algorithms [Fig. 3(a)]; then, the blood vessel information was manually extracted from the proton density-weighted images [Fig. 3(b)]; next, multi-modal image fusion algorithms integrated these structural datasets [Fig. 3(c)] resulting in a preliminary 3D wrist model [Fig. 3(d)]; the original MRI images have a slice thickness of 2 mm and a slice spacing of 0.725 mm, causing a stair-step effect [Fig. 3(d)]; therefore, an adaptive spatial smoothing protocol was applied to address it [Fig. 3(e)]; finally, finite element meshing (FEM) transformed the optimized volumetric data into original DW [Fig. 3(f)].

#### 2.1.2 Generation of scaled target DWs

**Fig. 4:**
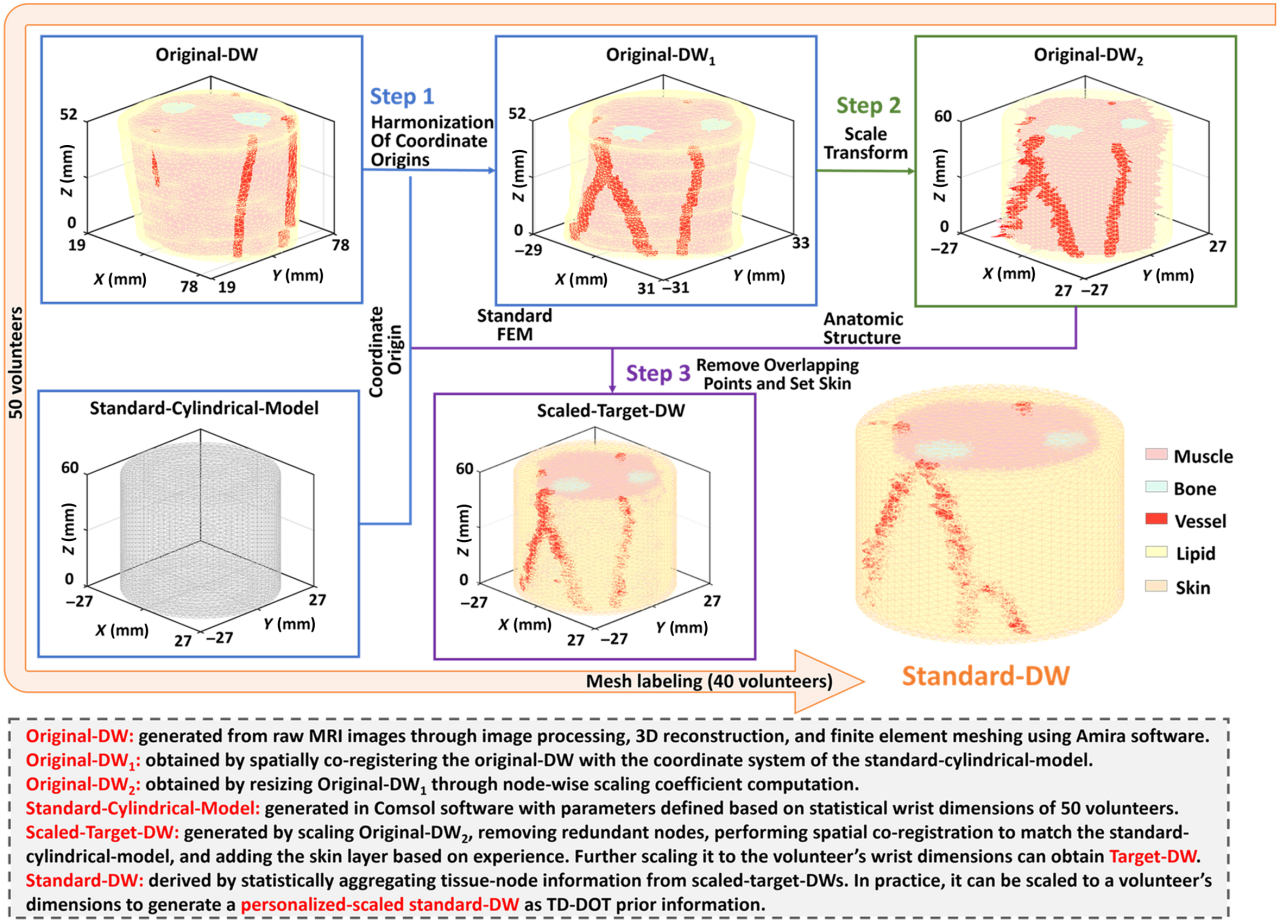
Flowchart of scaled target DW generation.

Individual variations in wrist size and surface morphology result in heterogeneity among the FEM of the original DWs, making direct statistical analysis of tissue-related nodes impractical. Therefore, geometric normalization is a crucial step in constructing a statistical standard anatomical model. In this study, the reference geometry was simplified to a cylindrical structure (standard cylindrical model) based on the following rationale. Measurements from 50 volunteers [Fig. 2(c)] showed that the aspect ratios (major-to-minor axis ratios) of the cross-sections at the ulnar styloid level and at 1/9 and 2/9 of the proximal forearm were 1.27±0.07, 1.15±0.08, and 1.13±0.09, respectively, indicating that the cross-sectional shape gradually transitions from an elliptical to an approximately circular form. Meanwhile, Fig. 2(d) shows that the radii of the cross-sections at these three levels are very similar, further supporting the approximation of the wrist geometry as a cylinder. Based on the averaged measurement data, the parameters of the standard cylindrical model were defined as follows: height = 60 mm (corresponding to 2/9 of the forearm length) and radius = 27 mm (mean radius across the measured levels). The original wrist models were then proportionally scaled according to the flowchart illustrated in Fig. 4 to generate scaled target DWs, as detailed below.

Step 1: Spatial co-registration of the original DW with the standard cylindrical model coordinate system yielded transformed original ${\mathrm{DW}}_{1}$ as follows:
  $$F(r_{n}^{\left(O1\right)},C_{n}^{\left(O1\right)},\Omega_{e}^{\left(O1\right)}):\;\{\begin{array}{l} r_{n}^{\left(O1\right)}=[x_{n}^{\left(O1\right)},y_{n}^{\left(O1\right)},z_{n}^{\left(O1\right)}],n=1,2,\ldots ,N^{\left(O\right)}\\ C_{n}^{\left(O1\right)}=c,c\in \{1,2,\ldots ,C\}\\ \Omega_{e}^{\left(O1\right)}=[i_{e},j_{e},k_{e},l_{e}],i_{e},j_{e},k_{e},l_{e}\in \{1,2,\ldots ,N^{\left(O\right)}\} \end{array},$$ (1)
  where O denotes the original type, $r_{n}^{\left(O1\right)}$ is the spatial coordinate of the n’th node, $C_{n}^{\left(O1\right)}$ is the n’th node’s tissue type (c=1∼4 represents muscle, bone, blood vessel, and lipid, respectively), $\Omega_{e}^{\left(O1\right)}$ is the e’th tetrahedral element, and $i_{e}$, $j_{e}$, $k_{e}$, and $l_{e}$ are the corresponding nodes.
Step 2: Node-wise scaling coefficient computation for original ${\mathrm{DW}}_{1}$ to original ${\mathrm{DW}}_{2}$ transformation: axial feature lines were constructed by connecting all nodes to their orthographic projections along the Z-axis. These lines intersected triangular facets on original ${\mathrm{DW}}_{1}$’s lateral surfaces, generating geometrically constrained feature nodes. For each intersection, the nearest centroid was designated as the target control point, with distance (from control points to their Z-axis projections) forming a matrix $R^{\left(O\right)}$. Planar scaling coefficients in XOY dimensions were calculated as $m_{xoy}=R^{\left(O\right)}/R^{\left(cy\right)}=[m_{xoy,1},m_{xoy,2},\cdots ,m_{xoy,N^{\left(O\right)}}]$, where $R^{\left(cy\right)}$ denotes the radius of the standard cylindrical model. The Z-direction scaling coefficient is $m_{z}=H^{\left(O\right)}/H^{\left(cy\right)}$ with $H^{\left(cy\right)}$ representing the reference height. The node set of original ${\mathrm{DW}}_{2}$ was subsequently derived via coordinate transformation: $r_{n}^{\left(O2\right)}=[m_{xoy,n}x_{n}^{\left(O1\right)},m_{xoy,n}y_{n}^{\left(O1\right)},m_{z}z_{n}^{\left(O1\right)}]$.
Step 3: Redundant nodes removal and skin labeling to generate the scaled target DW: non-uniform scaling of original ${\mathrm{DW}}_{1}$ nodes caused overlapping nodes in original ${\mathrm{DW}}_{2}$. To resolve this, original ${\mathrm{DW}}_{2}$ was first spatially co-registered with the standard cylindrical model $r^{\left(cy\right)}$ ($R^{\left(cy\right)}=27\;\mathrm{mm}$, $H^{\left(cy\right)}=60\;\mathrm{mm}$, number of nodes $N^{\left(cy\right)}=30,173$) through minimum-distance nodal mapping. Based on empirical values,^28^ the superficial 1.5 mm was then designated as skin, resulting in the scaled target DW: $F(r_{n}^{\left(cy\right)},C_{n}^{\left(\mathrm{st}\right)},\Omega_{e}^{\left(cy\right)})$, where $C_{n}^{\left(st\right)}=c$, c∈{1,2,…,C}, c=1∼5 represents muscle, bone, blood vessel, lipid, and skin; cy denotes the standard cylindrical model type and st denotes the scaled target DW type.

#### 2.1.3 Mesh labeling

**Fig. 5:**
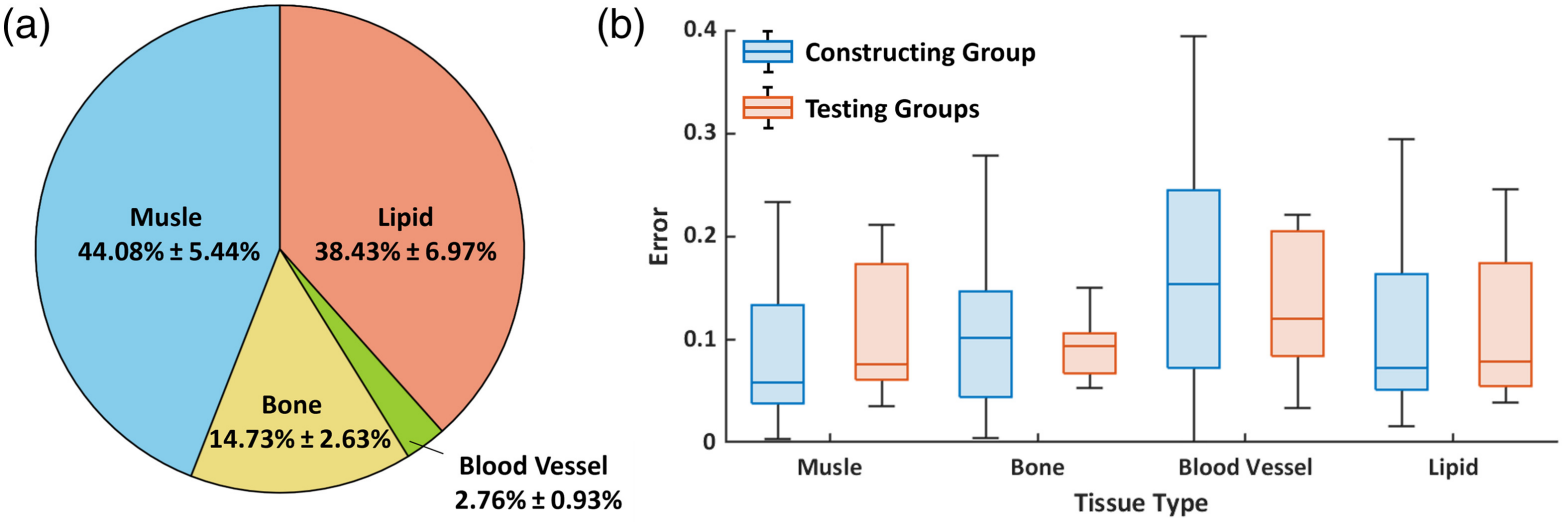
Tissue and result analysis. (a) Averaged tissue proportions among 50 volunteers. (b) Error between standard DW and scaled target DWs, defined as the difference in node counts between the tissue in the standard DW and the scale target DW, divided by the total number of nodes. The box represents the interquartile range (25th to 75th percentile), the line inside the box indicates the median, the whiskers extend to the minimum and maximum values, and points beyond them are shown as outliers.

Figure 5(a) demonstrates the compositional profile of wrist tissues in an MRI image across all 50 scaled target DWs: large-sized tissues (muscle, bone, and lipid) were classified via the maximum probability method, whereas the blood vessels were determined using the coordinate averaging method to address the incomplete node information due to the minute structure, completing the standard DW template:

$$F(r_{n}^{\left(cy\right)},C_{n}^{\left(S\right)},\Omega_{e}^{\left(cy\right)}),$$ (2)

where $C_{n}^{\left(S\right)}=\overset{¯}{c}$, $\overset{¯}{c}=1∼5$ represents muscle, bone, blood, vessel, lipid, and skin, respectively.

The maximum probability method is a statistical approach that assigns to each node of the standard DW the tissue type with the highest occurrence probability across all models. The coordinate averaging methodology: first, given that retained blood vessels in each standard target DW exhibited consistent directional orientations and morphological characteristics, the blood vessel with its centroid position closest to the group average (called BV) was designated as the representative vascular morphology for the standard DW. Subsequently, the mean volumetric measurement of blood vessels across all standard target DWs was computed, and the element count of BV was adjusted to match this average volume, thereby generating standardized blood vessel-type nodes that constitute the standard DW. As the skin layer thickness is consistent between the standard DW and the scaled target DWs (both 1.5 mm superficially), subsequent error analysis was performed only for the remaining tissues and the overall wrist. The error was defined as the difference in node counts between the tissue in the standard DW and the scale target DW, divided by the total number of nodes in the standard DW. As shown in Fig. 5(b), the standard DW exhibited an average error of 11.48% relative to the construction group and 12.39% relative to the testing group, indicating that it can reasonably capture wrist anatomical features and demonstrates practical applicability.

### 2.2 Multimodal Optical Characterization of Wrist Tissues

To acquire the optical properties of wrist tissues, this work implemented region-based TD-DOT for 3D reconstruction of deep tissue optical properties, complemented by the SFDI method to quantify the optical properties of the superficial skin layer.

#### 2.2.1 Region-based TD-DOT for deeper tissue

The generalized pulse spectrum technique (GPST) method was employed for TD-DOT reconstruction.^29^ This approach first applies a Laplace transform to the TD signals and then performs model inversion in the complex frequency domain. To simultaneously enhance computational efficiency and disentangle absorption–scattering interactions, two real transformation factors ($p_{1}$ and $p_{2}$) are introduced.^30^^,^^31^ Compared with the full-time-resolved schemes, this feature-based approach is both computationally simpler and more robust.^32^^,^^33^

Data calibration: to adapt forward calculations, a reference measurement is introduced to eliminate the system impulse response function (IRF) and couple variations effects in TD-DOT data.^34^^,^^35^ The size-match reference phantom is measured identically to target measurements, with IRFs relative eliminated through target/reference transformation ratios, generating calibrated data as follows:

Γ^(ξd,ζs,pi)=Γ^Tar(ξd,ζs,pi)Γ^Ref(ξd,ζs,pi)×ΓM(ξd,ζs,pi)=ΓTar(ξd,ζs,pi)×I(ξd,ζs,pi)ΓRef(ξd,ζs,pi)×I(ξd,ζs,pi),i=1,2, (3)

where ${\hat{\Gamma }}_{\mathrm{Tar}}(\xi_{d},\zeta_{s},p_{i})$ and ${\hat{\Gamma }}_{\mathrm{Ref}}(\xi_{d},\zeta_{s},p_{i})$ are the Laplace-transformed data of the target and reference measurements, respectively; $\Gamma_{\mathrm{Tar}}(\xi_{d},\zeta_{s},p_{i})$ and $\Gamma_{\mathrm{Ref}}(\xi_{d},\zeta_{s},p_{i})$ are the Laplace-transformed data of the target and reference measurements, respectively, with the IRF removed. $\Gamma_{M}(\xi_{d},\zeta_{s},p_{i})$ is the model-calculated transformed data regarding the geometry and the optical properties of the reference phantom; $I(\xi_{d},\zeta_{s},p_{i})$ is the IRFs of all source-detector pairs in the TD-DOT system; $\xi_{d}$ (d=1,…,D) and $\zeta_{s}$ (s=1,…,S) denote the illuminating and detecting positions, respectively.

Optical inversion: the modified version of GPST is employed for cost-effectiveness, which is based on the discretized Laplace-transformed diffusion equation regarding the intermediate variable $O(r_{n},p_{i})$^29^

$$\delta \Gamma (p_{i})=J(p_{i})O(p_{i}),$$ (4)

where $\delta \Gamma (p_{i})=[(\xi_{1},\zeta_{2},p_{i}),(\xi_{2},\zeta_{1},p_{i}),\ldots ,(\xi_{D},\zeta_{S},p_{i})]$ numerates the perturbed data for all the source-detector pairs, with $\delta \Gamma (\xi_{d},\zeta_{s},p_{i})=\overset{⌢}{\Gamma }(\xi_{d},\zeta_{s},p_{i})-\Gamma_{M}(\xi_{d},\zeta_{s},p_{i})$; $O(p_{i})=[O(r_{1},p_{i}),O(r_{2},p_{i}),\ldots ,O(r_{N},p_{i}){]}^{T}$ lists the intermediate variable $O(r,p_{i})$ at all the discrete nodes, and $J(p_{i})$ is the Jacobian matrix with its entry calculated as

$$J(\xi_{d},\zeta_{s},r_{n},p)=-\alpha \sum_{r_{n}\in \Omega_{e}}\langle \Gamma (\xi_{d},r_{n},p_{i})\Phi (r_{n},\zeta_{s},p_{i})\rangle V(\Omega_{e}),$$ (5)

where $\Gamma [\xi_{d},r_{n},p_{i}]$ and $\Phi (r_{n},\zeta_{s},p_{i})$ are the green function regarding the boundary flux and inner fluence distribution calculated for the current optical properties, respectively; $V(\Omega_{e})$ is the volume of the e’th element $\Omega_{e}$ containing node $r_{n}$; α=1/4 for the used tetrahedral element; ⟨·⟩ denotes the mean within $\Omega_{e}$.

With the intermediate variable $O(r_{n},p_{i})$ calculated at the transform factors $p_{1}$ and $p_{2}$, the absorption and reduced scattering coefficients can be separated as follows:^29^

$$\left\{ \begin{array}{l} \delta \mu_{a}(r_{n})=\frac{\left[\mu_{a}(r_{n})c+p_{2}]O(r_{n},p_{1})-[\mu_{a}(r_{n})c+p_{1}]O(r_{n},p_{2}\right)}{p_{1}-p_{2}}\\ \delta \mu_{s}^{\prime }(r_{n})=\frac{[O(r_{n},p_{1})-O(r_{n},p_{2})]\mu_{s}^{\prime }(r_{n})c}{p_{1}-p_{2}} \end{array}.\right.$$ (6)

In the implementation, Eqs. (4) and (6) are solved simultaneously within the iterative framework. To address the inherent ill-posedness, algebraic reconstruction techniques (ART) are employed during each iteration to ensure stable numerical solutions.^29^

Based on the personalized-scaled standard DW, this work assumed the optical homogeneity within tissue constituents; thereby, reducing voxel-based reconstruction to “coarse-grained” parameter estimation of constituent optical properties,^15^ including muscle, bone, blood vessel, and lipid. The Jacobian matrix accordingly assumes the following form, systematically transformed from voxel-based matrices through a tissue region-voxel mapping matrix

$$\overset{˜}{J}=\mathrm{JG},$$ (7)

where $\overset{˜}{J}$ represents the Jacobian matrix based on tissue regions; G is of size $N^{\left(cy\right)}\times C$, with its term given as

$$G_{n,c}=\{\begin{array}{cc} 1 & \text{if}\;r_{n}\in \Omega_{c,}\\ 0 & \text{otherwise} \end{array},$$ (8)

where $\Omega_{c}$ is the c’th tissue region of the personalized-scaled standard DW.

#### 2.2.2 SFDI for superficial skin

The region-based DOT approach relies on the diffusion equation, which inadequately models light transport in superficial skin layers. Moreover, the typical source–detector separation of 21.20 to 84.78 mm, optimized for deep-tissue signals, further reduces sensitivity to superficial skin properties. Together, these factors limit the accuracy of superficial skin measurements. As a complement, we employed an *in vivo* SFDI approach based on the Monte Carlo (MC) model, validated in previous studies.^17^^,^^18^^,^^36^^,^^37^

This methodology employed sinusoidal wide-field structured illumination to acquire diffuse reflectance images ($\Gamma_{\mathrm{Tar}}(x,y)$) of superficial skin layers. A single-pixel imaging framework based on two-dimensional discrete cosine transform was applied to convert the raw data into spatial-frequency domain diffuse reflectance profiles (${\hat{\Gamma }}_{\mathrm{Tar}}(\lambda ,f_{x})$),^17^^,^^37^^–^^39^ where λ represents the operational wavelengths employed for spectral quantification of tissue optical properties, and $f_{x}$ denotes the spatial frequency of the illumination pattern. Spatial frequencies of 0 and $0.2\;{\mathrm{mm}}^{-1}$ were chosen for superficial skin characterization^40^^,^^41^: $0\;{\mathrm{mm}}^{-1}$ provides stable reflectance representing bulk tissue properties, whereas $0.2\;{\mathrm{mm}}^{-1}$ is more sensitive to superficial variations. Combined, they enable more accurate characterization of skin optical properties. In practical applications, a reference-based calibration framework is employed to enhance measurement accuracy,^42^ where a phantom with known optical properties undergoes the same acquisition and processing as biological tissue to correct system-induced errors, such as illumination non-uniformity and detector response inconsistencies.

A fast lookup-table framework incorporating bounded interval optimization was implemented for optical properties extraction.^43^ At each spatial frequency, the tissue surface diffuse reflectance varies monotonically with the absorption coefficient ($\mu_{a}$) and reduced scattering coefficient ($\mu_{s}^{\prime }$). The effective search range of optical properties is determined based on the measured maximum and minimum reflectance values, with the lookup conducted within this optimized range. For each property set, the theoretical diffuse reflectance is compared with the experimental measurements using the least-squares distance, and the combination of properties with the minimum error is selected as the final reconstruction result, enabling quantification of the optical properties.^18^^,^^37^ The optical properties–reflectance database, generated via the MC–Hankel hybrid method,^44^ encompassed physiologically relevant ranges for human tissues in visible-near infrared regimes: $0.001\;{\mathrm{mm}}^{-1}\leq \mu_{a}\leq 0.5\;{\mathrm{mm}}^{-1}$, $\Delta \mu_{a}=0.001\;{\mathrm{mm}}^{-1}$; $0.5\;{\mathrm{mm}}^{-1}\leq \mu_{s}^{\prime }\leq 1.5\;{\mathrm{mm}}^{-1}$, $\Delta \mu_{s}^{\prime }=0.01\;{\mathrm{mm}}^{-1}$.

## 3 Methodological Validations

The performance of the DOI methodology is systematically evaluated through a comprehensive experimental protocol. Initially, as the SFDI method has been validated in previous studies,^17^^,^^36^ simulation experiments were conducted solely to assess the efficacy and accuracy of the GPST-region-based TD-DOT methodology. Subsequently, both TD-DOT and SFDI systems were developed, and phantom experiments were conducted to verify their performance. Finally, *in vivo* experiments involving 50 volunteers were carried out, which not only demonstrated the feasibility, effectiveness, and potential clinical applicability of the proposed method but also established a statistically significant standard optical DW.

### 3.1 Simulative Validation

To evaluate the feasibility of using a standard DW template for TD-DOT reconstruction, we conducted numerical simulations under specified conditions. This section describes the data generation, the geometric adaptation of the standard DW template, and the results.

#### 3.1.1 Data generation

**Fig. 6:**
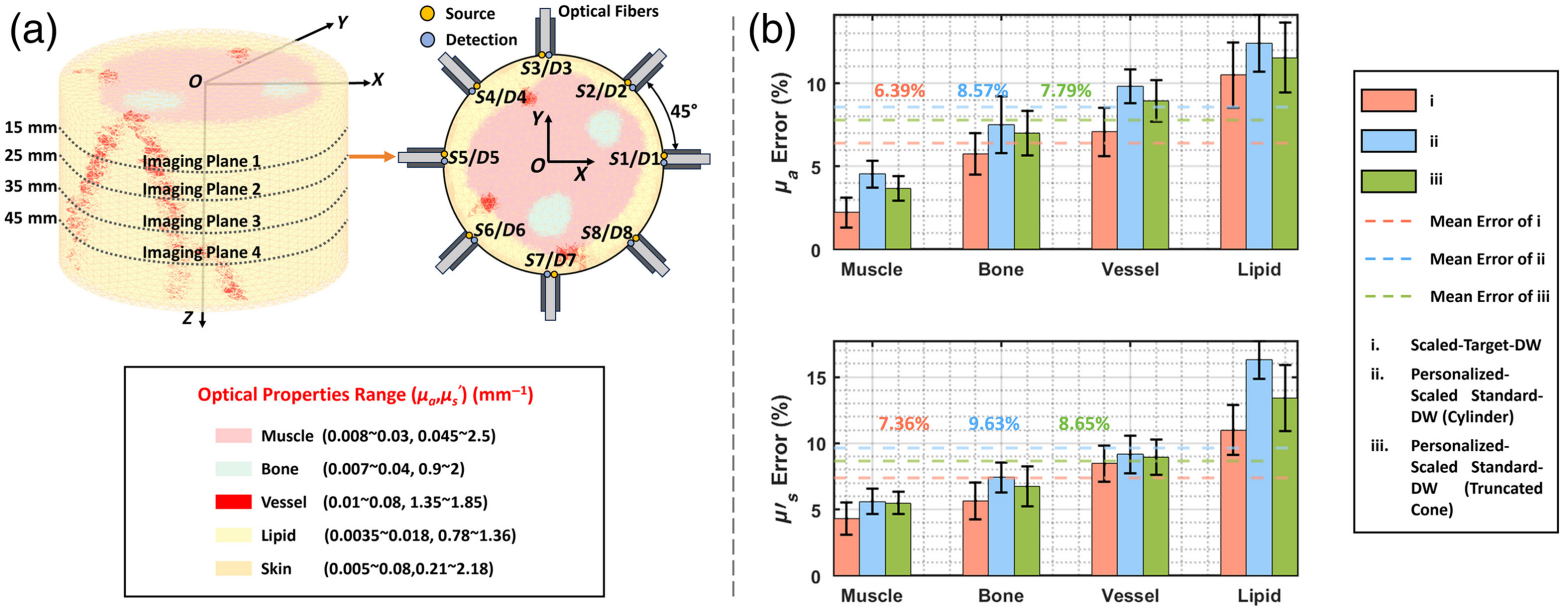
Simulation validation. (a) Experiment configuration. (b) Reconstruction results.

Simulation validation was performed using the scaled target DWs of the test group, with 25 sets of optical properties. These properties were generated using a random number generator in MATLAB. Specifically, based on the reported ranges shown in Fig. 6(a),^24^^,^^25^^,^^45^^–^^51^ the upper and lower limits of the optical properties were determined for each tissue type. Subsequently, uniformly distributed random values were generated within these ranges, resulting in 125 independent $\left(\mu_{a},\mu_{s}^{\prime }\right)$ combinations (i.e., 25 sets for each of the 5 tissue types). The source-detector array spanned 15 to 45 mm along the model’s Z-axis, comprising four measurement layers with eight confocal illumination-detection points each. The detector separation range was set from 21.20 to 84.78 mm. Each measurement cycle illuminated one position while sequentially acquiring data from seven detectors. The MC method^52^^,^^53^ was employed to simulate TD-DOT measurements. To better approximate the performance of the actual instrument, the simulated data were convolved with the experimentally measured IRF at the wavelength of 830 nm, and Gaussian noise $\Gamma_{i}=\Gamma_{i}(1+Q_{G}10^{-{\mathrm{SNR}}_{i}/20})$ was added, where $\Gamma_{i}$ denotes the i’th measurement. ${\mathrm{SNR}}_{i}={\mathrm{SNR}}_{\min}•\sqrt{\Gamma_{i}/\min(\Gamma )}$ is the signal-to-noise ratio (SNR), and ${\mathrm{SNR}}_{\min}$ was maintained at 20 dB to approximate experimental conditions. Furthermore, additional simulations were performed to assess the impact of IRF FWHM variations on reconstruction accuracy in TD-DOT (see Supplementary Material).

#### 3.1.2 Personalized scaling of standard DW

The 2/9 forearm length $H^{\left(T\right)}$ of the volunteer was measured to calculate the Z-direction scaling coefficient $m_{z1}=H^{\left(T\right)}/H^{\left(cy\right)}$. The radius $R^{\left(T0\right)}$ at the 1/9 layer of the forearm was used to calculate the XOY plane scaling coefficient $m_{xoy1,n}=R^{\left(T0\right)}/R^{\left(cy\right)}$. Then, the personalized-scaled standard DW (cylinder) can be acquired by registering the standard DW.

Although there is little difference in wrist circumference among the proximal/distal layers in the selected samples, considering the size diversity within the DOT applicable population necessitated additional truncated-cone modeling. The Z-direction scaling coefficient is the same as the personalized-scaled standard DW (cylinder): $m_{z2}=m_{z1}=m_{z}$. Ulnar styloid process radius ($R^{\left(T1\right)}$) defined the superior surface geometry, and the Z-axis coordinates of the nodes were $z^{\left(T1\right)}$. The 2/9 forearm-level wrist circumference ($R^{\left(T2\right)}$) established the inferior surface geometry, and the Z-axis coordinate of nodes is $z^{\left(T2\right)}$. Thus, the XOY plane scaling coefficient was obtained as follows:

$$m_{xoy2,n}=(\frac{z_{n}^{\left(T\right)}-z^{\left(T1\right)}}{z^{\left(T2\right)}-z^{\left(T1\right)}}(R^{\left(T2\right)}-R^{\left(T1\right)})+R^{\left(T1\right)})/R^{\left(cy\right)}.$$ (9)

Finally, the personalized-scaled standard DW’s nodes were calculated as follows:

$$r_{n}^{\left(T\right)}=[m_{xoy\varphi ,n}x_{n}^{\left(cy\right)},m_{xoy\varphi ,n}y_{n}^{\left(cy\right)},m_{z}z_{n}^{\left(cy\right)}],\;n=1,2,\ldots ,N^{\left(cy\right)},\;\varphi =1,2.$$ (10)

In addition, due to the characteristics of TD-DOT imaging, the skin and subcutaneous lipid layers are modeled as a single layer during reconstruction.

#### 3.1.3 Results

Figure 6(b) shows that the mean reconstruction errors of $\mu_{a}$ were 6.39% (scaled target DW), 8.57% (cylinder), and 7.79% (truncated cone), respectively, with corresponding $\mu_{s}^{\prime }$ mean errors of 7.36%, 9.63%, and 8.65% for scaled target DW, cylinder, and truncated cone, respectively, indicating relatively high reconstruction accuracy.^54^^,^^55^ It is noteworthy that the mean errors among the three models are very similar. This result indicates that the standard DW provides sufficient anatomical information for TD-DOT, thereby substantially reducing the dependence on time-consuming MRI-based individualized models. These findings establish a theoretical foundation for subsequent clinical translation studies.

Accordingly, this reconstruction approach was applied to obtain *in vivo* optical properties from 50 volunteers, establishing the standard optical DW via Eqs. (2) and (11)

$$O_{c,\lambda }^{\left(S\right)}=[\mu_{a,c,\lambda },\mu_{s,c,\lambda }^{\prime }],\;\lambda \in \{670\;\mathrm{nm},830\;\mathrm{nm},905\;\mathrm{nm}\},$$ (11)

where $O_{c,\lambda }^{\left(S\right)}$ is the statistical *in vivo* optical properties of each tissue at the λ nm wavelength, $\mu_{a,c,\lambda }$ is the absorption coefficient, and $\mu_{s,c,\lambda }^{\prime }$ is the reduced scattering coefficient.

### 3.2 Experimental Validation

We developed three-wavelength TD-DOT and SFDI systems for the phantom validation of the methodology, as well as later *in vivo* optical measurement of the population’s wrists.

#### 3.2.1 System composition

**Fig. 7:**
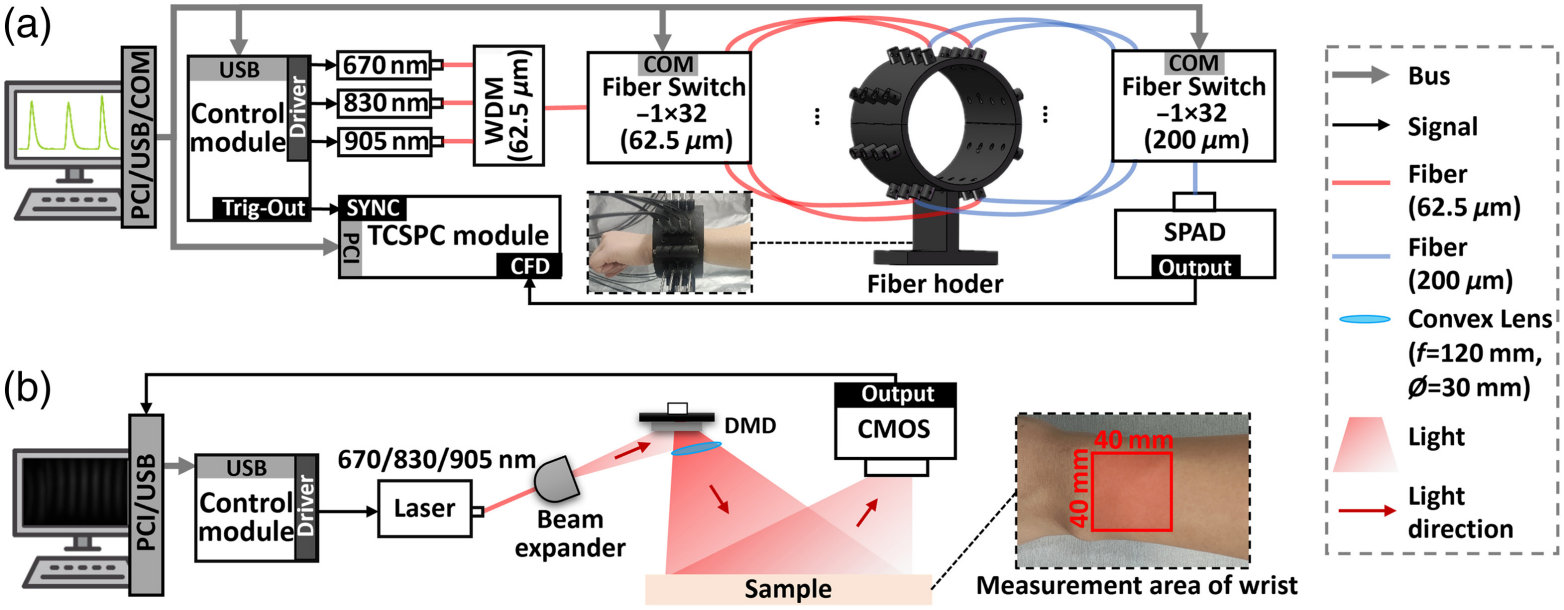
System schematic. (a) TD-DOT. (b) SFDI. PCI, peripheral component interconnect; USB, universal serial bus; COM, communication port; TCSPC, time-correlated single-photon counting; SYNC, synchronization signal; CFD, constant-fraction discriminator; WDM, wavelength-division multiplexer; SPAD, single-photon avalanche diode; DMD, digital micro-mirror device; CMOS, complementary metal-oxide-semiconductor.

The TD-DOT system is designed for simultaneous data collection at the three wavelengths with adaptive integration time capability, as shown in Fig. 7(a). The system employs a multi-channel controller (PDL-828, PicoQuant, Berlin, Germany) to synchronously drive picosecond pulsed diode lasers at the three wavelengths (LDH-P-670/830/905, PicoQuant). Laser outputs are combined through a wavelength-division multiplexer (WDM-14P-670-830-905, OZ Optics, Carp, Canada) into a single source fiber (62.5-μm core diameter, 0.22 NA) then distributed via a source-side optical switch (CETC-FSW1×32-MM-62.5, CETC, Beijing, China). The fiber arrangement on phantoms or wrist surfaces replicates the simulation configuration shown in Fig. 6(a). Detection fibers (200-μm core diameter, 0.22 NA) interface with a detection-side optical switch (CETC-FSW1×32-MM-200, the same manufacturer). Transmitted photons are detected by a single-photon avalanche diode (SPCM-AQRH-14-FC, Excelitas, Pittsburgh, Pennsylvania, United States) and processed through a time-correlated single photon counting module (TCSPC) (SPC130, Becker & Hickl, Berlin, Germany).

In addition, an adaptive data acquisition protocol was implemented to optimize both the SNR and temporal efficiency. For each source–detector pair, the system continuously monitors the peak counts of the time-point spread function (TPSF) relative to a predefined threshold (3000 counts). Once this threshold is reached, the corresponding integration time is recorded, and the acquisition proceeds to the next channel. If the threshold is not reached within a maximum integration time of 3 s, the system automatically advances. The TCSPC time bin width was set to 12.2 ps, which enables high depth resolution while maintaining a relatively short measurement time. The overall system sampling rate can be approximated as the total number of source-detector pairs divided by the total acquisition time. Compared with conventional multi-wavelength schemes using fixed integration times, this adaptive strategy substantially reduces the total acquisition time while maintaining a high SNR.

The schematic of the SFDI system employed in this work is illustrated in Fig. 7(b). The system utilizes the same model of multi-channel controller and picosecond pulsed diode lasers as those implemented in the TD-DOT system. After beam expansion and redirection, the laser passes through a digital micromirror device (DMD, DLP4500-0.45-WXGA, Texas Instruments, Dallas, Texas, United States) to generate SFDI structured illumination patterns, is further expanded by a convex lens (H-K9L, VisionFly, Bengaluru, India), and finally detected by a complementary metal-oxide-semiconductor (CMOS) camera (CC215MU-Quantalux-sCMOS, Thorlabs, Newton, New Jersey, United States). According to the methods reported in the literature,^56^ the DMD output axis was tilted 3 deg relative to the optical platform normal to reduce reflections and diffraction artifacts, whereas spatial averaging over multiple projection patterns minimized speckle. These measures preserved the integrity of the wide-field sinusoidal patterns in SFDI and effectively suppressed coherence-induced noise.

#### 3.2.2 Experimental characterization of TD-DOT

This section presents a comprehensive evaluation of the TD-DOT system, including its IRF, temporal stability, and ability to accurately retrieve optical properties in homogeneous media.^57^

**Fig. 8:**
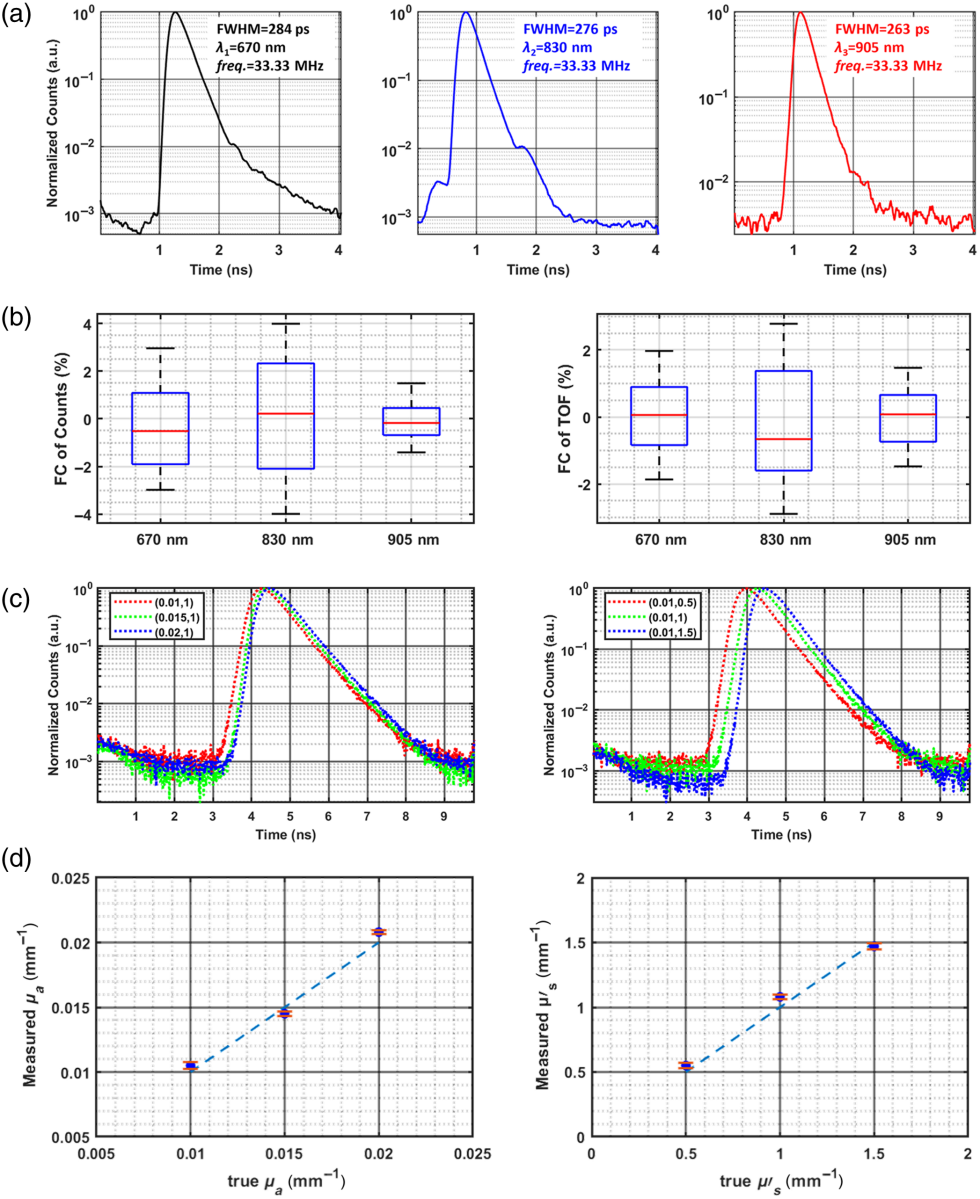
Experimental characterization of the TD-DOT system. (a) IRFs at 670, 830, and 905 nm. (b) Coefficients of variation of photon counts and mean time-of-flight. (c) Normalized time point spread function (TPSF) curves at 830 nm for different phantoms, with increasing $\mu_{a}$ and constant $\mu_{s}^{\prime }$ (left) and increasing $\mu_{s}^{\prime }$ with constant $\mu_{a}$ (right). (d) Comparison of average recovered $\mu_{a}$ and $\mu_{s}^{\prime }$ with their nominal values. Error bars represent standard deviations over 10 repetitions.

The typical IRFs of the system are shown in Fig. 8(a), corresponding to wavelengths of 670, 830, and 905 nm. They were obtained by directly coupling the collection and injection fibers while keeping the overall count rate of the detection module below 10% of the laser repetition rate (33.33 MHz) to minimize signal distortion due to pile-up effects.^58^ Each curve represents an integration time of 10 s to enhance the SNR. IRF broadening arises mainly from the laser pulse width, fiber propagation, and detector response, with all IRFs in this system having full width at half maximum (FWHM) below 300 ps, within a reasonable range.

The temporal stability of the instrument is critical for long-term TD-NIRS measurements. To evaluate system performance, a continuous 3-h test, including the warm-up period, was conducted on a solid phantom ($\mu_{a}=0.01\;{\mathrm{mm}}^{-1}$, $\mu_{s}^{\prime }=1\;{\mathrm{mm}}^{-1}$). The source–detector separation was 10 mm apart, and data were acquired every 3 min with a 1-s integration time. System stability was evaluated by examining variations in the fluctuation coefficient (FC, FC(i)=[In(i)−mean(In(i))/mean(In(i)]×100%, where In(i) denotes the measured photon count/time-of-flight for each trial, and mean[In(i)] denotes the average of multiple measurements). As shown in Fig. 8(b), after a 30-min warm-up, FC of count for all three laser wavelengths remained below ±3.6%, and FC of mean flight time was within ±2.5%, demonstrating the excellent temporal stability of the TD-DOT system.

To further assess the system’s ability to extract optical properties in homogeneous media, liquid phantoms were prepared using India ink (absorbing agent), intralipid (scattering agent), and distilled water.^59^ Experiments were divided into two groups: in the first, $\mu_{s}^{\prime }$ was fixed at $1\;{\mathrm{mm}}^{-1}$ while $\mu_{a}$ was set to 0.01, 0.015, and $0.02\;{\mathrm{mm}}^{-1}$; in the second, $\mu_{a}$ was fixed at $0.01\;{\mathrm{mm}}^{-1}$ while $\mu_{s}^{\prime }$ was set to 0.5, 1, and $1.5\;{\mathrm{mm}}^{-1}$. Reflectance measurements were performed with a 10-mm source–detector separation, 3-s integration time, and 10 repetitions per sample. As shown in Fig. 8(c), variations in $\mu_{a}$ and $\mu_{s}^{\prime }$ clearly affect the normalized TPSF curves. Both properties were estimated via least-squares fitting using the standard diffusion model,^60^ yielding results in excellent agreement with the set values [Fig. 8(d)], demonstrating the system’s ability to differentiate phantoms with varying optical properties. Only data at 830 nm are presented, as results were similar across the three wavelengths.

### 3.3 Phantom Experiment

As mentioned in Sec. 2.2.1, the reference phantom plays a critical role in system calibration by eliminating the IRF effects. Given the predominance of muscle tissue in wrist anatomy, optical properties were derived from *in vivo* measurements of forearm muscle regions [Fig. 10(a)] in 10 volunteers using the fitting method.^61^ The results show that at 670, 830, and 905 nm, the mean $\mu_{a}$ of muscle was 0.0134, 0.0122, and $0.0115\;{\mathrm{mm}}^{-1}$, respectively, with corresponding $\mu_{s}^{\prime }$ values of 1.3975, 1.2860, and $1.1572\;{\mathrm{mm}}^{-1}$, respectively. These values exhibit concordance with established literature.^24^^,^^25^^,^^45^

**Fig. 9:**
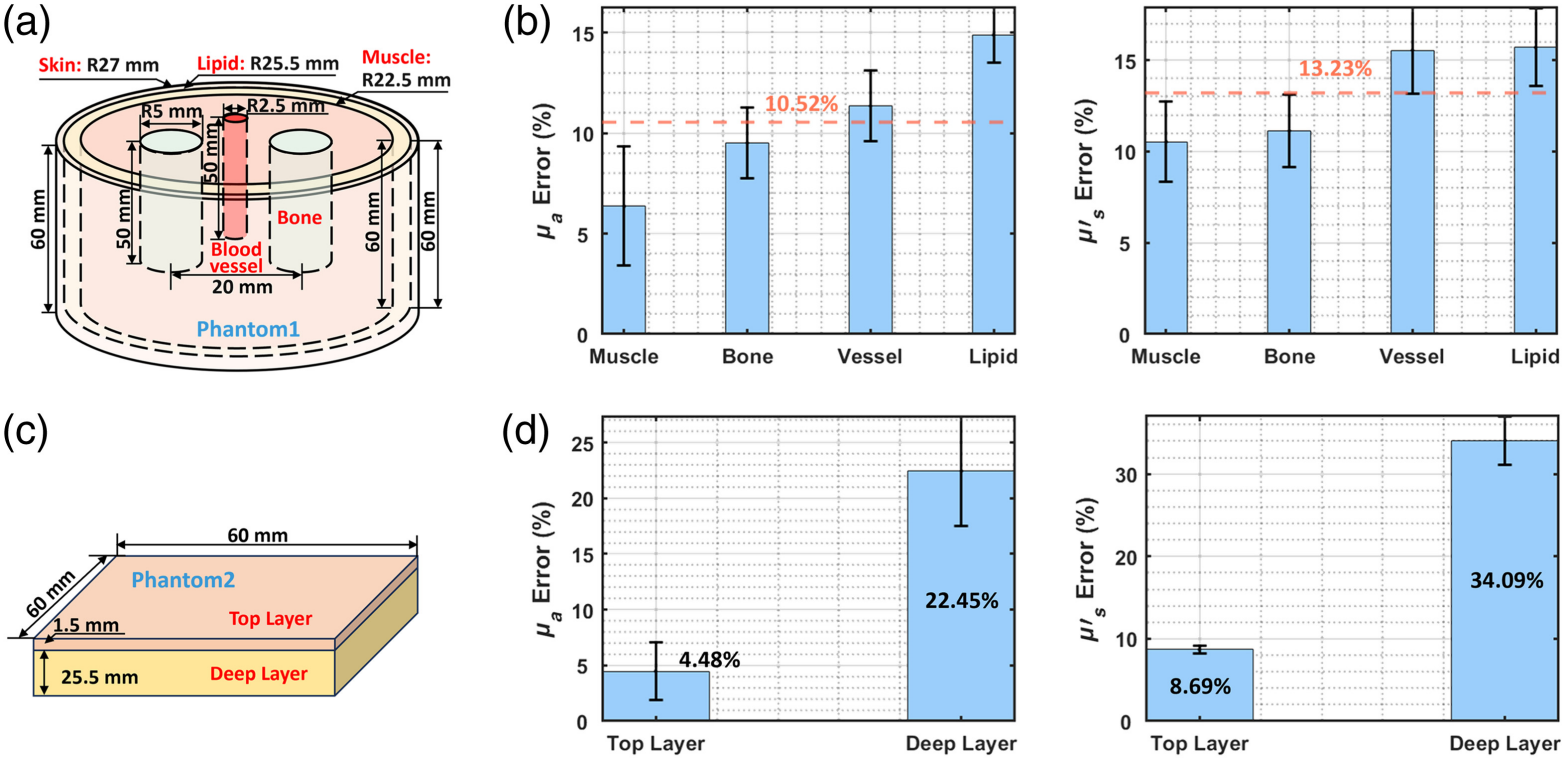
Phantom experiment. (a) Wrist phantom. (b) Results of TD-DOT. (c) Skin phantom. (d) Results of SFDI.

The geometry of phantom1 [Fig. 9(a)] was determined through anthropometric analysis of 50 volunteers, incorporating two critical parameters: forearm length (2/9 ratio) and radius averages at three anatomical levels (ulnar styloid, 1/9 forearm length, and 2/9 forearm length). This analysis produced cylindrical phantoms (height: 60 mm) with radii incrementing from 22 to 32 mm in 2 mm steps. The phantoms shown in Figs. 9(a) and 9(c) are solid, with epoxy resin as the matrix, India ink as the absorber, and titanium dioxide as the scatterer.^62^ Molds for each phantom module were 3D-printed according to the designed dimensions. Phantom 1 was fabricated by first casting and curing deep structures such as vessels and bone, followed by sequential casting and curing of muscle, fat, and skin layers. Phantom 2 was prepared by casting a 25.5-mm-thick bottom layer first, followed by a 1.5-mm-thick top layer. The optical properties used were based on the simulation settings in Fig. 6(a) and were divided into five groups evenly. TD-DOT reconstruction showed mean errors of 10.52% for $\mu_{a}$ and 13.23% for $\mu_{s}^{\prime }$ [Fig. 9(b)]. SFDI experiments on phantom 2 demonstrated superior accuracy, with top layer $\mu_{a}$ and $\mu_{s}^{\prime }$ errors of 4.48% and 8.69%, respectively [Fig. 9(d)]. These results collectively validate the effectiveness of the reconstruction strategies and system performance for both TD-DOT and SFDI. It should be noted that the primary objective of the SFDI method is to accurately determine the optical properties of the superficial skin tissues. In the model design, the deeper tissue layer mainly serves as a diffusive background to provide sufficient optical thickness for photon propagation. Although the estimation errors of the deep-layer optical properties are relatively higher, this does not compromise the quantitative accuracy of the superficial optical properties or the overall goal of this work. As mentioned above, the optical information of the deeper tissues was complemented by the proposed region-based TD-DOT approach.

### 3.4 *In Vivo* Experiments

**Fig. 10:**
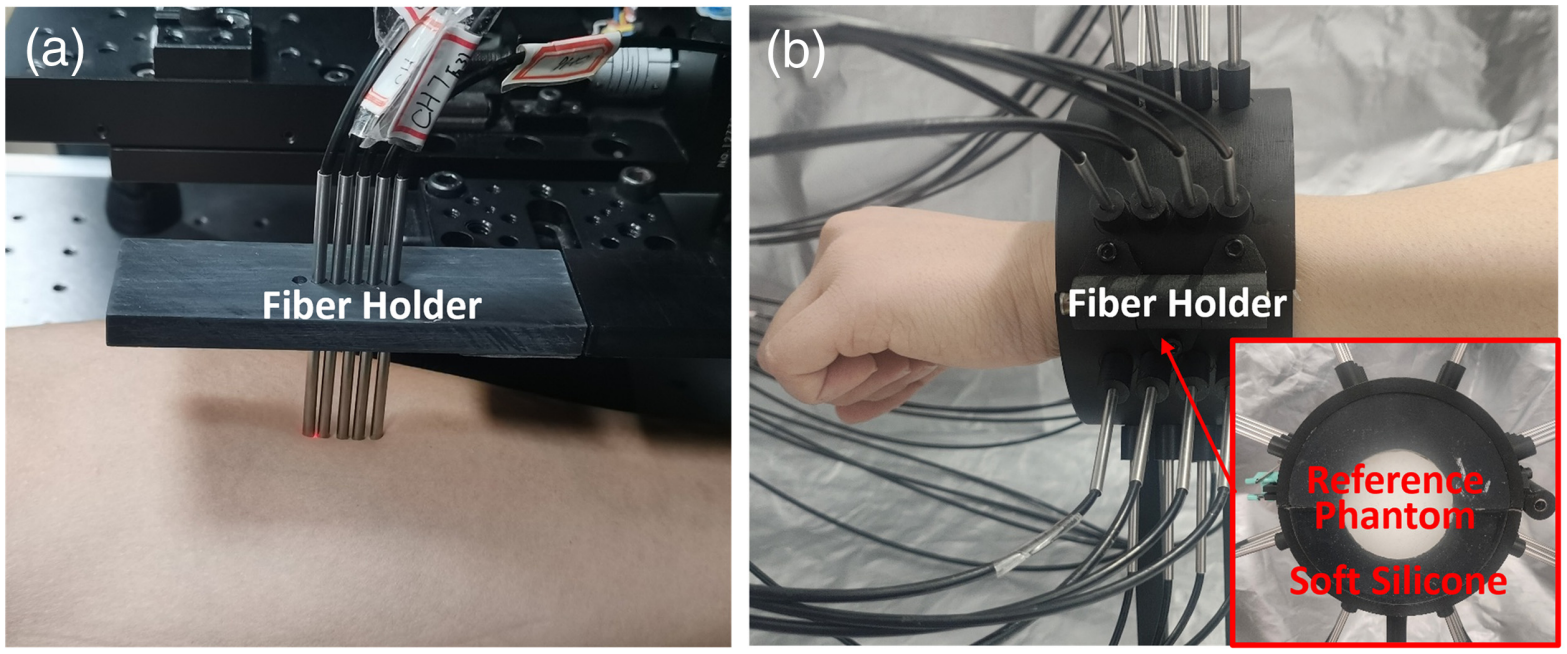
*In vivo* experiments. (a) Measurement positions. (b) Posture and reference phantom.

As shown in Fig. 10(b), TD-DOT uses a measurement posture similar to MRI, with the fiber array coupled to the wrist via highly absorptive soft silicone. Fibers were kept in close contact with the skin to ensure stable optical signal acquisition. During reconstruction for the testing group, three models were sequentially implemented: the target DW, a personalized-scaled standard DW (cylinder), and a truncated cone variant, to evaluate performance differences. For the total dataset, only the personalized-scaled standard DW (cylinder) was employed.

**Fig. 11:**
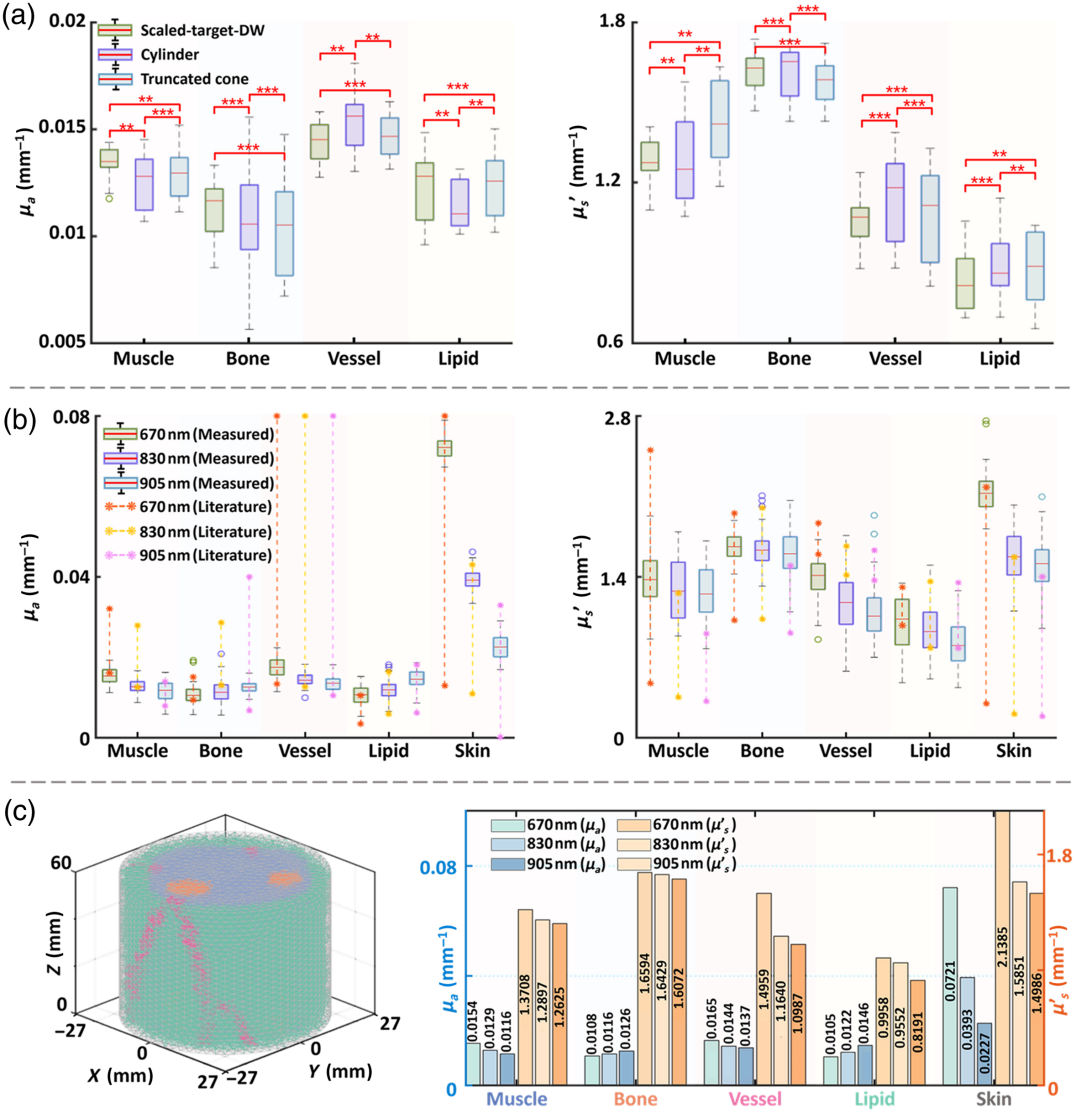
Results of *in vivo* experiment. (a) Results of testing group (10 volunteers) comparison among different models at 830 nm. Correlation classification: ***$R^{2}\geq 0.95$; **$0.90\leq R^{2}<0.95$; *$R^{2}<0.90$. (b) Results of personalized-scaled standard DW (cylinder) of total datasets (50 volunteers). (c) Standard optical DW.

Figure 11(a) presents a statistical comparison of optical properties reconstructed at 830 nm using three models in the testing group. Coefficient of determination ($R^{2}$) indicates high consistency among the models, strongly suggesting that the personalized-scaled standard DW can effectively replace MRI-based individualized wrist models (scaled target DW) while maintaining accurate *in vivo* measurements. Moreover, the minimal difference between cylindrical and truncated cone models indicates that a simplified cylindrical geometry is sufficient for most practical applications.

Figure 11(b) compares the optical properties obtained in this work with literature values. The results show that most tissue optical properties fall within reported ranges, though minor discrepancies exist, such as the $\mu_{s}^{\prime }$ of vessels and $\mu_{s}^{\prime }$ of bone at 905 nm.^24^^,^^25^^,^^45^^–^^48^ These differences may arise from variations in individual tissue properties, measurement methods, and optical transport models. Notably, this work employed *in vivo* TD-DOT measurements, whereas literature values were obtained using CW-NIRS, frequency-domain methods, double-integrating sphere method, or *ex vivo* samples.^24^^,^^25^^,^^45^^–^^48^^,^^50^^,^^51^ Subsequently, a standard optical DW [Fig. 11(c)] was constructed by averaging tissue optical properties at 670, 830, and 905 nm from 50 volunteers, producing the anatomically refined and optically representative model.

## 4 Discussion and Conclusion

This work established a statistically based standard optical DW by integrating MRI and DOI. Using MRI data from 40 volunteers, a population-averaged standard DW was first constructed and then scaled to individual wrist dimensions to generate a personalized-scaled standard DW. The model showed errors of 11.48% and 12.39% compared with target DWs in the construction and testing groups, respectively, supporting that population-based scaling can effectively capture wrist anatomy. Further simulations demonstrated reconstruction errors below 8.57% for $\mu_{a}$ and 9.63% for $\mu_{s}^{\prime }$, quantitatively validating the efficacy and precision of the proposed reconstruction approach.

To enhance practical applicability, reference phantoms were used to eliminate the IRF. After initial TD-DOT system validation, wrist phantom experiments further evaluated TD-DOT and SFDI performance, followed by *in vivo* wrist measurements. Reconstructions using scaled target DW and personalized-scaled standard DW (cylindrical and truncated cone) models yielded highly consistent optical properties [Fig. 11(a)], with results across the database generally matching literature values [Fig. 11(b)]. Averaging region-specific optical properties from 50 volunteers at 670, 830, and 905 nm established a standard optical DW [Fig. 11(c)]. Notably, the multi-wavelength dataset reveals wavelength-dependent trends in optical properties, enhancing its reference value and broadening compatibility with diverse optical biosensing technologies.

It should be noted that the standard optical DW developed in this work was constructed based on population-averaged data, successfully establishing a reference framework that integrates typical anatomical characteristics and optical properties distributions of the wrist. Although the model demonstrates strong representativeness within the East Asian population aged 20 to 60 years, its applicability can be further extended to broader age ranges and skin tones. The cylindrical approximation adopted in the current model has proven robust for modeling; however, the fact that the ulnar styloid level exhibits an elliptical cross-section (aspect ratio = 1.27) suggests that future work could further improve individual adaptability through geometric optimization. In terms of tissue modeling, the model effectively captures population-level trends through a statistically derived fat-to-muscle ratio and geometric scaling strategy, though there remains room for improvement in representing individual variations in tissue composition. Future work will incorporate samples covering diverse skin tones and age groups, and integrate physiological parameters such as body mass index to build correlation-based models that enhance universality and biological realism. Although the diffusion equation in TD-DOT has limitations in modeling low-scattering tissue (e.g., lipid-rich tissues),^63^ the standardized methodological framework established in this work provides a foundation for integrating advanced photon transport models such as MC simulations^52^^,^^53^ and for incorporating dynamic optical measurements. Overall, this work contributes a population-based standard optical DW that bridges the gap between idealized homogeneous optical assumptions and realistic anatomical complexity, offering both theoretical and practical value for the design of customizable, high-reliability health-sensing systems and advancing the development of optical technologies toward personalized medical applications.

## Supplementary Material

10.1117/1.JBO.30.12.126003.s01

## Data Availability

Data underlying the results presented in this paper may be obtained from the corresponding authors upon reasonable request and under a licensing agreement.
