# Supplementary material for: Development of a statistically standardized optical digital wrist model through integrated MRI-diffuse optical imaging methodology
Source: J Biomed Opt. 2025 Dec 3;30(12):126003. doi: 10.1117/1.JBO.30.12.126003 (PMC12674630; doi:10.1117/1.JBO.30.12.126003)
Supplement: Supplementary file 1 [file JBO_030_126003_SD001.docx]

**Supplemental Material**

*Impact of IRF FWHM Variations on Reconstruction Accuracy in TD-DOT*


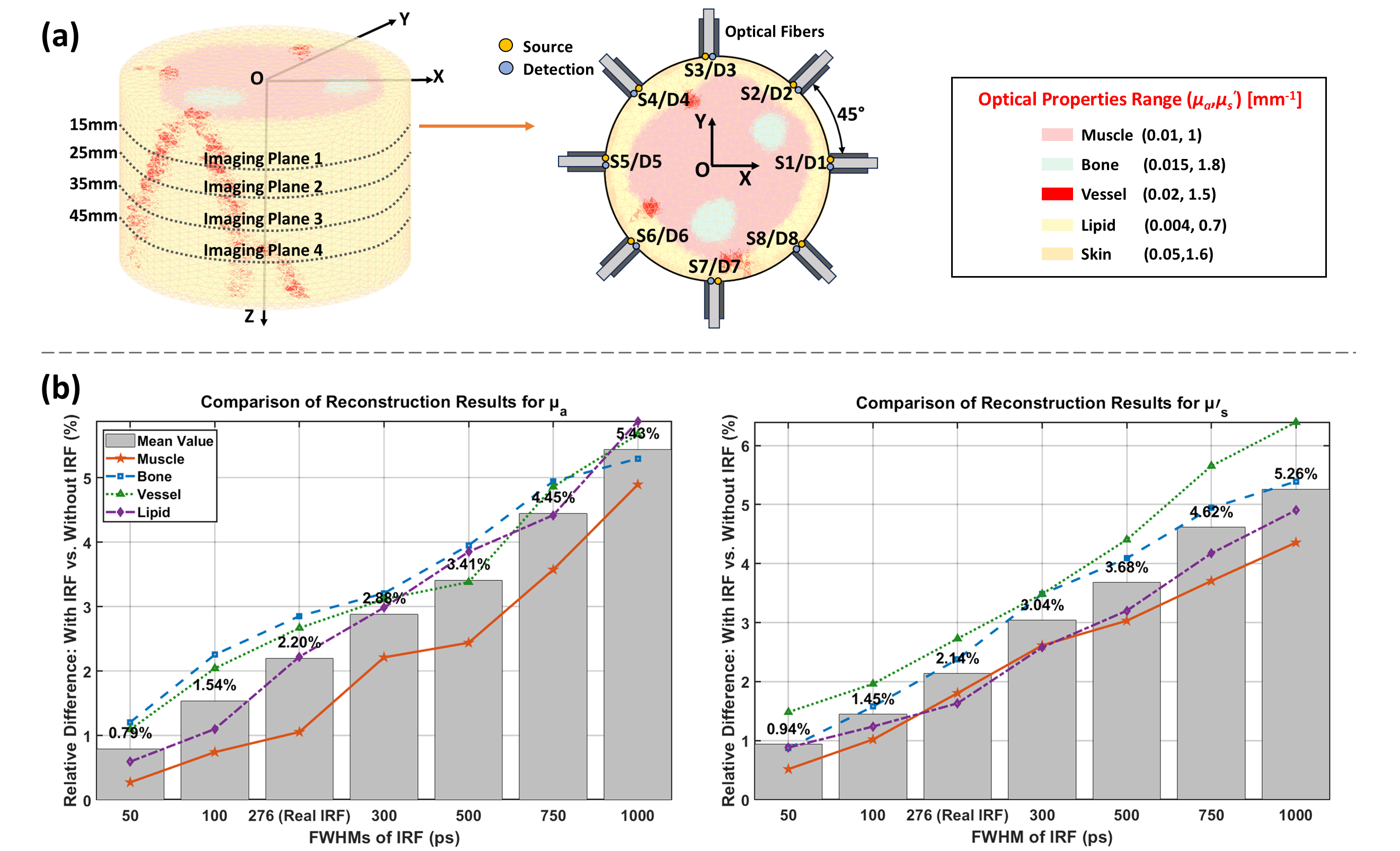


**Fig. S1** Simulation validation: (a) experiment configuration; (b) relative differences between reconstruction results with different FWHM of IRF and without IRF.

In time-domain diffuse optical tomography (TD-DOT) systems, the instrument response function (IRF) acts as a low-pass filter that attenuates the high-frequency temporal components of the temporal point spread function (TPSF), thereby potentially affecting the accuracy of optical property reconstruction. To quantitatively evaluate the impact of the IRF on reconstruction results, this study systematically analyzed the effect of different IRF widths, characterized by the full width at half maximum (FWHM), on reconstruction performance.

Based on the experimentally measured IRF, a series of IRFs with FWHMs of 50 ps, 100 ps, 276 ps (corresponding to the measured IRF at 830 nm), 300 ps, 500 ps, 750 ps, and 1000 ps were generated by broadening and compressing the original measured IRF. Simulations were performed using the scaled-target DWs from the testing group, whose optical properties and source-detector configuration are shown in Fig. S1(a). Each IRF was convolved with the TPSF obtained from Monte Carlo simulations, followed by the addition of 20 dB Gaussian noise. The optical properties were then reconstructed using the region-based TD-DOT method with a personalized-scaled standard-DW (cylinder).

Figure S1(b) shows the relative differences between the reconstruction results obtained under different IRF FWHMs and those without IRF convolution. As the FWHM increased, the reconstruction errors for both absorption (*μ_a_*) and reduced scattering coefficients (*μ_s_^’^*) gradually rose. Notably, when the FWHM was below 750 ps, all relative differences remained within 5%, indicating good robustness of the proposed method in this range. For the experimental system used in this study, the measured IRF at 830 nm (FWHM = 276 ps) resulted in mean relative differences of 2.20% and 2.14% for the *μ_a_* and *μ_s_^’^*, respectively. Moreover, the measured IRFs at 670 nm (FWHM = 284 ps) and 905 nm (FWHM = 263 ps) also fell within this stable range. Overall, the results demonstrate that the proposed reconstruction method maintains high accuracy and stability under the IRF conditions of the current TD-DOT system.
